# Discovering Hominins - Application of Medical Computed Tomography (CT) to Fossil-Bearing Rocks from the Site of Malapa, South Africa

**DOI:** 10.1371/journal.pone.0145340

**Published:** 2015-12-18

**Authors:** Jacqueline S. Smilg, Lee R. Berger

**Affiliations:** 1 Department of Diagnostic Radiology, Charlotte Maxeke Johannesburg Academic Hospital, Johannesburg, South Africa; 2 Evolutionary Studies Institute, University of the Witwatersrand, Johannesburg, South Africa; 3 Department of Science and Technology/National Research Foundation Centre of Excellence in Palaeosciences, University of the Witwatersrand, Johannesburg, South Africa; University of Oxford, UNITED KINGDOM

## Abstract

In the South African context, computed tomography (CT) has been used applied to individually prepared fossils and small rocks containing fossils, but has not been utilized on large breccia blocks as a means of discovering fossils, and particularly fossil hominins. Previous attempts at CT imaging of rocks from other South African sites for this purpose yielded disappointing results. For this study, 109 fossil- bearing rocks from the site of Malapa, South Africa were scanned with medical CT prior to manual preparation. The resultant images were assessed for accuracy of fossil identification and characterization against the standard of manual preparation. The accurate identification of fossils, including those of early hominins, that were not visible on the surface of individual blocks, is shown to be possible. The discovery of unexpected fossils is reduced, thus lowering the potential that fossils could be damaged through accidental encounter during routine preparation, or even entirely missed. This study should significantly change the way fossil discovery, recovery and preparation is done in the South African context and has potential for application in other palaeontological situations. Medical CT imaging is shown to be reliable, readily available, cost effective and accurate in finding fossils within matrix conglomerates. Improvements in CT equipment and in CT image quality are such that medical CT is now a viable imaging modality for this palaeontological application.

## Introduction

Fossils offer tangible evidence of the past and are important for the study of the prehistory of life on Earth. They are typically formed through diagenesis and object replacement by a wide variety of minerals and elements [1]. In the Cradle of Humankind (COH) World Heritage site [2], fossils from the Plio-Pleistocene era are usually found in dolomitic limestone caves, encased in hard calcified sediments. These are often referred to as breccias or calcified clastic matrix [1,3]. This breccia encases bones and varies in its hardness and density. Whilst the strength of the rock has protected the fossils, the density of the rock also makes extraction of the fossils from their surrounding matrix difficult.

Traditionally fossils have been manually prepared from their encasing matrix, or prepared using methods involving acetic or other acids. This involves both mechanical or chemical extraction which is often time consuming and potentially damaging to the fossils themselves [3]. Furthermore, the search for fossils immediately below the surface of the rock being prepared (beyond those made visible by the extraction process or by natural erosion), is typically a haphazard affair, traditionally completely reliant upon the skills of the preparator and random chance. It is therefore often very difficult to completely clean or reconstruct fossils without in some way damaging them. Compounding the difficulty of preparation, fossils are also often incomplete or filled with calcified matrix [4]. These manual methods of preparation are, in addition, destructive to the surrounding matrix and information not recognized during the process may be permanently lost. Even advances in preparation using automated techniques suffer these same problems [5]. Traditional methods of fossil preparation often severely limit research and due to the fact that many fossils of interest to palaeontologists are exceedingly rare, other methods have been examined to allow better visualization and interpretation, whilst at the same time potentially preserving the fossil material and associated matrix. Advances in computer technology, software and the quality of X-rays machines have seen an increase in the use of these X-ray based modalities to “virtually” prepare fossils [6]

X-rays have been commonly used for medical diagnosis since their discovery in 1895 by Wilhelm Röntgen. The use of X-rays in palaeontology dates back to 1896, when Brühl [7] in Berlin and Lemoine [8] in Paris first used X- rays to image fossils. Branco [9] produced the first published work on the use of X-rays for fossil imaging in 1906, followed by Jaekel in 1921, Mautz in 1929 and Lehmann in 1934, who investigated the marine fossils of the Hünsruck slate with X-ray images [9]. Wilhelm Stürmer, a chemical physicist and radiologist at Siemens Corporation, combined Lehmann’s experience with his own interest in palaeontology and developed new methods of examining the Hunsrück fossils using X-rays [10]. Consequently he produced detailed radiographs of unprepared slates, using soft X-rays (25–40 KV) and stereoscopic exposures, combined with high-resolution films and image processing. These showed some detail of soft tissue not revealed by the conventional techniques that Lehmann had used.

Historical attempts at X-ray imaging of fossil bearing matrix has typically been reported as producing poor results, thought to be due to the density of the material, inclusions in the matrix and lack of resolution of images produced by the equipment used [4]. A major limitation of conventional X-rays was a 2 dimensional image of 3 dimensional structures, resulting in superimposition of all structures in the path of the X- ray beam [4]. Conventional X- rays did not have good differential tissue resolution and thus lacked the ability to provide detailed information about internal structures. Additionally, in the case of fossils, mineralized tissues have similar abilities to absorb X-rays and thus X-ray images were not able to detect difference between these preserved tissues and between them and the surrounding matrix.

Computed Tomography (CT) was invented in 1972 by Godfrey Hounsfield [11]. Compared to conventional X-rays, CT provides higher resolution and cross sectional as well as 3D images. CT additionally has a number of advantages over traditional X- rays. Its greatest benefit perhaps is that it can distinguish between substances of differing densities better than conventional radiographs.

CT was introduced 43 years ago, but its use for palaeoanthropological applications has still to be fully exploited. In 1991, Grine stated that “*the employment of CT in palaeontology is potentially even more problematic because diagenetic factors that may affect the mineralization of fossil teeth can only but add to the factors that can confound the use of CT*” [12].

It however has been recognized that CT was able to acquire interior information non-destructively from irreplaceable fossil specimens [13,14]. In 1984 Conroy applied CT scanning to a mammalian cranium and after that success he used CT to scan hominin fossils [15]. More detailed studies would follow, with Conroy and others using CT in the analysis of fossil hominin skulls and fossil dental enamel thickness amongst others [12,16,17]. Morphometric studies focusing on such structures as mandibular cross sections [18,19]; femora [20] and labyrinthine structures [21–23] obtained from CT scans were well received, while those on enamel thickness [12] were questioned [24,25].

In palaeoanthropological applications, CT has been used mostly to assess skulls.[16,26–30] But other bones have been examined by CT, including temporal bones, mandibles, femurs and other post cranial elements [18,31–37]. As CT imaging has improved due to software improvements and engineering improvements to equipment, high resolution, modern CT has also been found to be very useful for studying the delicate internal structure of smaller anatomical structures such as the para nasal sinuses, the inner ear and the microanatomy of teeth [21,38–42].

Rapid advances in CT in the 21st century, that run parallel with significant advances in computing technology, as well as software improvements, have made high resolution fossil imaging and reconstruction viable due to expanded CT number scales and the use of special image reformatting software that has provided qualitative and quantitative 3D imaging [6]. Additionally, helical CT—introduced in 1989 [27]—is now significantly better than conventional CT, with higher energy (mAs) capabilities [43]. Thus the combination of software and hardware advances has offered considerably greater potential for the application of CT in palaeontology.

These advances in technology have made the use of CT in the analysis of prepared fossils common place [44]. Most of the CT work to date has been performed on prepared or partially prepared specimens. However, the application of CT to matrix that potentially contains fossils has lagged behind these many advances in the visualization and study of prepared fossils. Very little work has been done to image, via CT, large fossil- bearing matrix conglomerates fresh out of the field. This has, in part, been due to the demand to apply these new technologies to fossils that have already been prepared, or are currently under study, and also to the fact that many matrices that potentially contain fossils have not been previously considered suitable for such imaging, based upon earlier non-rigorous and occasional tests. Furthermore, as palaeontologists and palaeontological technicians have not typically been trained in the interpretation of CT images, the perception seems to have existed that it would be difficult or near impossible to identify fossils still encased in anything but minute amounts of rock. Specifically, previous attempts to use CT to image rocks with potential palaeoanthropological interest has resulted in generally poor results and little effort has been made to apply these methods in the 21^st^ century [4].

Advances in CT technologies, combined with the discovery of sites and localities with denser matrix, containing fewer inclusions have, however, shown promising results for the application of CT technologies to unprepared sediments [45]. A study by Bollinger and colleagues [46] describes the use of multi detector CT in locating, identifying and examining fossil remains of 3 crocodilians embedded in hard shale whilst Rahman and colleagues [47] saw the combination of computer science and the study of past life as creating “*an incredibly exciting field”*.

In February 2009, a breccia block discovered at Malapa, was found to contain the diaphysis of a humerus (later to be assigned to *A*. *sediba* MH1). In April 2009, this block was undergoing manual preparation when a portion of a maxilla was uncovered. This maxilla appeared to belong to an early hominid. Due to its potential importance and prior to further preparation, better visualization was sought of what might be hidden from the preparator’s view. On 21 April 2009, the first CT scans of the Malapa material were performed. The visualized maxillary bone was in fact part of an entire juvenile cranium (MH1). The quality of visualization obtained from the CT images gave the first hint that the Malapa calcified clastic sediments were particularly suitable to X-ray penetration.

This discovery also laid the groundwork for the present research and a process of scanning of unprepared blocks was begun.

The aim of this research was to determine the viability of medical CT scanning for use in the identification and characterization of fossils within unprepared matrix blocks from the fossil hominin bearing site of Malapa in the Cradle of Humankind World Heritage site, against the gold standard of traditional block preparation using manual techniques to expose fossils. If successful, such methods could prove cost effective and preserve and protect material, while allowing greater success in discovering and recognizing important fossils.

## Materials

The site of Malapa lies to the north of Johannesburg, South Africa in an area known as the Cradle of Humankind (COH)—a UNESCO World Heritage Site declared due to its important hominin fossil—bearing localities [2]. In the late 19^th^ and early 20^th^ century, lime miners traversed this area in search of mineable lime. The lime miners test blasted many sites in their search for economically mineable lime, leaving behind many localities that are only slightly damaged by such activities. The site known as “Malapa” is one such area. It represents a de-roofed cave that has been exposed by years of erosion [3]. After some initial limited blasting, the miners appear to have abandoned further mining activity. Even this limited mining activity, however, left large rocks strewn across the surface of the site. It is some of these rocks that have been collected from the site and taken to what was then the Institute of Human Evolution (IHE) and is now the Evolutionary Studies Institute (ESI) at the University of the Witwatersrand for analysis and examination in this study. The blocks are variable in size. For many of the blocks, their exact context within the fossil deposit on the site is known and recorded, for others, the exact location of recovery is not known, only their presence within the miners’ dumps at the site are known as well as their association with the site. The blocks for scanning were chosen due to the presence of visible bone on the exterior of the blocks or due to their potential to yield fossils as determined by their position on the site. 109 blocks were scanned and analyzed. Each block was assigned a “B” number as well as a “UW88” site number for identification purposes.

A medical CT scanner at the Charlotte Maxeke Johannesburg Academic Hospital (CMJAH)—Somatom Definition AS 40 from Siemens (Erlangen, Germany)—was used for the scanning of the 109 blocks. For post processing and interrogation of the medical CT scan data, the images were stored on compact disc (CD) and Digital Imaging and Communication in Medicine (DICOM) images were assessed on an Apple MacBook (Mac OS X version 10.5.8) with OsiriX software (version 3.5.1–64 bit). The CT reader is a diagnostic Radiologist, trained in radiological human anatomy and cross sectional imaging.

## Methods

Excavations at the site of Malapa governed by an excavation permit as follows:

Issuing Body: South African Heritage Resources Agency (SAHRA)

Permit Holder: Lee R. Berger

Permit ID: 1946

Case ID: 6407

Validity: 15 January 2015–31 January 2018

The fossil blocks are under the custodial care of the Evolutionary Studies Institute (ESI) at the University of the Witwatersrand, Johannesburg, South Africa.

109 blocks from the Malapa site were scanned. Site/specimen numbers (designated by UW 88) and Block numbers (designated by “B” numbers) were assigned to each block. Preliminary visual identification was made on each block, prior to scanning, of any bone visible on the surface. This identification was done by technical staff of the IHE. Radiographers assisted with the production of the CT images.

CT scanning parameters were chosen–Table 1.

**Table 1:** CT Scanning parameters used for the scanning of fossil breccia.

Matrix size	512 x 512
Field of View (FOV)	Individualized according to size of rock
Slice thickness	1 mm
Pitch	0.45
mAs	360
kVp	140

During reconstruction of the raw CT data, kernels are used to enhance spatial and contrast resolution. The kernel is a reconstruction parameter affecting image sharpness and noise by applying a specific mathematical algorithm that digitally filters the raw data during reconstruction. The authors experimented with different kernels, visually assessing the image for suitability of fossil identification. It was found that the H70h was overall best for the specimens scanned. This used a high resolution reconstruction kernel producing a sharper image, although greater noise. This kernel was found to improve bone/fossil visualization with edge enhancement and better spatial resolution. Interpretation of the CT images of the 109 blocks was done prior to block preparation and a colour code was assigned to each block to denote the findings—Table 2

**Table 2:** Colour assignment depicting CT findings.

Colour assigned to block	CT Findings
Red	Identifiable bone—probable hominin/primate
White	Identifiable bone—not hominin/primate
Yellow	Non identifiable bone or absence of bone

Following completed analysis of all 109 CT scans, representative blocks were prepared manually by preparators in the IHE. Due to the costly, time consuming nature of manual preparation, 44 blocks were chosen for this manual preparation, after communication between the scientists and the radiologist. Blocks were chosen using a combination of the colour coding assessment assigned from the CT analysis as well as the deemed importance of each block. The latter used visualized surface findings in combination with the deemed importance of the location at which the block was found on the site. The actual specimen findings following manual preparation of these 44 blocks were documented and correlation between the CT findings and the actual findings was made (Table 3).

**Table 3:** CT findings vs preparation findings.

UW Number	Block number	Scan venue	Priority	Surface ID	CT Findings	Volume cm^3^	Preparation findings	CT vs preparation findings
UW88-1316	B001	CMJAH	yellow	nil	nil	12304	Unidentified fragment	concordant
UW88-1342	B027	CMJAH	yellow	nil	fragments	862	Pelvic fragment	concordant
UW88-1365	B050	CMJAH	red	nil	Phalanx, long bone (tibia/ulna)	1451	Distal bovid metapodial, bovid metacarpal	minor variance
UW88-1368	B053	CMJAH	yellow	Bone fragments	fragments	285	Bone fragments	concordant
UW88-1376	B061	CMJAH	white	nil	Long bone (tibia)	2672		
UW88-1388	B073	CMJAH	white	nil	Femoral head	60	Bovid vertebra	discordant
UW88-1393	B078	CMJAH	yellow	nil	nil	238		
UW88-1396	B081	CMJAH	yellow	nil	Crystal, crushed bone	1030		
UW88-1421	B106	CMJAH	yellow	nil	Fragments	496	Fragments	concordant
UW88-1428	B113	CMJAH	yellow	nil	Long bone fragments	919	Carnivore metacarpal and long bone. Small mammal humerus and tibia	minor variance
UW88-1440	B125	CMJAH	red	nil	Fragments, possible scapula	418	Fragments. Parts of flat bone and long bone with cortical manganese	minor variance
UW88-1443	B128	CMJAH	yellow	Bovid antler fragment	Long bone fragment	41		
UW88-1456	B141	CMJAH	yellow	nil	fragments	782	1^st^ proximal phalanx, lateral end clavicle	minor variance
UW88-1462	B147	CMJAH	yellow	nil	nil	1297		
UW88-1472	B157	CMJAH	yellow	nil	fragments	1932	fragments	concordant
UW88-1476	B161	CMJAH	yellow	Long bone	Surface bone	897		
UW88-1479	B164	CMJAH	yellow	nil	Long bone	1700	Cervical vertebra bovid	discordant
UW88-1483	B168	CMJAH	yellow	nil	nil	2843	Tiny caudal vertebra bovid	discordant
UW88-1487	B172	CMJAH	yellow	Microfauna (tooth)	nil	682		
UW88-1491	B176	CMJAH	yellow	nil	nil	190		
UW88-1505	B190	CMJAH	yellow	fragment	fragment	2522	fragment	concordant
UW88-1506	B191	CMJAH	white	Rib fragment, micro fauna	Ulna bovid	473		
UW88-1523	B208	CMJAH	red	micro fauna	Malleolus/tibia ball joint	111		
UW88-1557	B242	CMJAH	yellow	nil	Long bone bovid	293		
UW88-1560	B245	CMJAH	red	nil	Hominin vertebra and rib	5387	Lumbar vertebra and rib from *A*. *sediba*. Body rib bovid II	concordant
UW88-1564	B249	CMJAH	yellow	Flow stone	Tubular bone	2720		
UW88-1566	B251	CMJAH	yellow	nil	Long bone fragment	1113		
UW88-1578	B263	CMJAH	yellow	Bovid rib fragment	Fragments	3797		
UW88-1586	B271	CMJAH	white	nil	Primate ribs, long bone fragments, artefact ++	4802		
UW88-1594	B279	CMJAH	yellow	nil	Fragments	5364		
UW88-1600	B285	CMJAH	yellow	Long bone fragment	fragments	3635		
UW88-1601	B286	CMJAH	yellow	nil	Long bone fragments	195	calcaneus	discordant
UW88-1613	B298	CMJAH	yellow	nil	Fragments	2269		
UW88-1629	B314	CMJAH	yellow	nil	Fragments	2224	Fragments	concordant
UW88-1631	B315b	CMJAH	yellow	nil	Fragments	7984		
UW88-1638	B322	CMJAH	yellow	Small mammal	nil	5491		
UW88-1650	B334	CMJAH	yellow	Snails, fly pupae, manganese, flowstone	fragments	7182		
UW88-1654	B338	CMJAH	white	nil	2 long bones (tibia/fibula)	792	Juvenile bovid tibia + fibula shaft fragments	concordant
UW88-1656	B340	CMJAH	white	fragments	Ribs/long bones	2987	Ribs + long bones	concordant
UW88-1658	B342	CMJAH	yellow	nil	Fragments	1362		
UW88-1670	B354	CMJAH	white	nil	Fragments, mandible piece	1782	Bovid III mandible ramus + fragments	concordant
UW88-1687	B371	CMJAH	white	nil	Long bone	1833		
UW88-1691	B375	CMJAH	white	nil	Ribs articulating with vertebrae, long bone, artefact ++	4315	5 x Articulated sub adult bovid vertebrae, with 2 ribs. Possible primate ulna	concordant
UW88-1695	B379	CMJAH	white	Pupae	Complex bone shape, rib	4316	Canid mandible, ribs	minor variance
UW88-1704	B388	CMJAH	yellow	nil	Thin curved bone, cranial fragments, artefact ++	2356	Bovid phalanx and skull fragments	concordant
UW88-1705	B389	CMJAH	yellow	Rat mandible	Fragments	1237		
UW88-1718	B402	CMJAH	yellow	Fragments	nil	8733		
UW88-1728	B412	CMJAH	yellow	Fly pupae, rock fragments	Bone fragments	1274		
UW88-1729	B413	CMJAH	yellow	nil	nil	10087	nil	concordant
UW88-1733	B417	CMJAH	yellow	? Bird bone, insect damage	nil	3349		
UW88-1753	B437	CMJAH	yellow	? Rabbit tooth	Fragments	477		
UW88-1754	B438	CMJAH	yellow	Dolomite inclusion	Fragments bone	1092		
UW88-1758	B442	CMJAH	white	nil	Vertebral elements, ribs	3403	Bovid vertebrae and ribs	concordant
UW88-1762	B446	CMJAH	white	nil	Long bone -crushed	1286		
UW88-1769	B453	CMJAH	red	nil	Fragments	420	Crushed bone fragments	concordant
UW88-1781	B465	CMJAH	white	nil	Bovid vertebra	3656	Thoracic bovid III vertebra	concordant
UW88-1785	B469	CMJAH	white	Clay nodules	Flat bone, fragments	1395	Flat bone fragment	concordant
UW88-1789	B473	CMJAH	yellow	Fragments, micro fauna,? burrows	Fragments bone	3063		
UW88-1791	B475	CMJAH	yellow	Fly pupae	Fragments bone	2615		
UW88-1792	B476	CMJAH	yellow	? Burrows	Small cube-like objects	2537		
UW88-1793	B477	CMJAH	yellow	nil	nil	1370		
UW88-1799	B483	CMJAH	yellow	Fragments bone, quartz	Fragments bone	3547		
UW88-1806	B490	CMJAH	white	nil	Bone fragments, distal femur, ribs/flat bone	2778	Bovid II distal femur, rib fragments bovid III	concordant
UW88-1807	B491	CMJAH	white	Bovid mandible	Mandible with teeth	9625	Bovid mandible and teeth	concordant
UW88-1812	B496	CMJAH	yellow	nil	Fragments bone	1587	Bone fragments	concordant
UW88-1813	B497	CMJAH	yellow	Long bone shaft fragment	Unidentifiable bone	5739		
UW88-1816	B500	CMJAH	yellow	nil	Bone fragments	3419	Bone fragments	concordant
UW88-1822	B506	CMJAH	yellow	CaCO_3_ stalactite, snail shells	Fragments bone	1804	Fragment mammalian rib	minor variance
UW88-1833	B517	CMJAH	white	nil	Vertebral, rib and long bone fragments	4378		
UW88-1834	B518	CMJAH	yellow	Fragments	Fragments bone	3806		
UW88-1836	B520	CMJAH	yellow	nil	Fragments bone	5699		
UW88-1840	B524	CMJAH	yellow	? stone tools	nil	4384		
UW88-1845	B529	CMJAH	yellow	inclusion	Fragments bone	2155		
UW88-1853	B537	CMJAH	yellow	Rock inclusion, fly pupae	Fragments	1013		
UW88-1857	B541	CMJAH	yellow	Dolomite inclusion	Fragments bone	2786		
UW88-1860	B544	CMJAH	yellow	nil	Fragments bone	3564		
UW88-1862	B546	CMJAH	yellow	nil	Fragments bone	1891	Bone fragments	concordant
UW88-1863	B547	CMJAH	yellow	Rock flakes	Fragments bone	2180		
UW88-1870	B554	CMJAH	white	nil	Long bones, vertebra, rib	1712	Small cat pelvis in articulation with vertebra and femur	minor variance
UW88-1871	B555	CMJAH	yellow	Clay nodule	Fragments bone	1234		
UW88-1876	B560	CMJAH	red	nil	Long bone fragment	5817	Long bone fragments	concordant
UW88-1877	B561	CMJAH	yellow	nil	nil	2422		
UW88-1879	B563	CMJAH	yellow	Dolomitic inclusion, rib fragment	Fragments bone, artefact ++	2535		
UW88-1887	B571	CMJAH	yellow	nil	nil	1390		
UW88-1888	B572	CMJAH	yellow	Fly pupae	nil	1853		
UW88-1904	B588	CMJAH	white	nil	Rib fragment, metapodial	1164		
UW88-1905	B589	CMJAH	white	nil	Flat bone	1717	Flat bone	concordant
UW88-1906	B590	CMJAH	yellow	Rock flake, fly pupae, snail shell	Fragments	1005	Bovid 2^nd^ phalanx fragment	minor variance
UW88-1910	B594	CMJAH	white	Air pockets	Long bone fragment	1727		
UW88-1912	B596	CMJAH	yellow	nil	Long bone fragment, artefact++	4985	Bird tibia—proximal fragment	minor variance
UW88-1918	B602	CMJAH	yellow	? Burrows	Bone fragment	2444		
UW88-1925	B609	CMJAH	yellow	airspaces	Fragments	1305		
UW88-1932	B616	CMJAH	yellow	nil	Bone fragments	1064		
UW88-1943	B627	CMJAH	yellow	nil	nil	1310		
UW88-1951	B635	CMJAH	yellow	nil	Fragments	1010		
UW88-1954	B638	CMJAH	yellow	Fragments bone	Fragments bone	939		
UW88-1955	B639	CMJAH	red	? Burrows,? organics	Distal femur/proximal tibia	1365	Proximal tibia	concordant
UW88-1962	B646	CMJAH	yellow	nil	nil	1113		
UW88-1971	B655	CMJAH	yellow	nil	Bone fragments	457	Bone fragments	concordant
UW88-1972	B656	CMJAH	yellow	Microfauna, dolomite	Fragments	924		
UW88-1988	B672	CMJAH	yellow	Fly pupae	Bone fragments	2613		
UW88-1999	B683	CMJAH	white	Burrows	Pelvic fragment	827	Pelvic fragment	concordant
UW88-2008	B692	CMJAH	white	nil	Femur large bovid	944	Distal bovid femur	concordant
UW88-2014	B698	CMJAH	white	nil	Bone fragment	2094	Bovid bone fragments	concordant
UW88-2040	B724	CMJAH	yellow	Dolomite/quartz	Long bone fragments	2481		
UW88-2044	B728	CMJAH	yellow	nil	fragments	1329		
UW88-2015	B735	CMJAH	yellow	nil	Fragments	2258		
UW88-2053	B737	CMJAH	yellow	nil	Fragments	738	Fragments	concordant
UW88-2386	B1070	CMJAH	yellow	nil	nil	680		

## Results and Discussion

The surface findings, CT findings and manual preparation findings are tabulated in Table 3 for each block by site/specimen number and block number. The colour code assigned to each block following CT analysis is tabulated. 109 blocks were scanned and 44 manually prepared. The findings are recorded in Table 3.

**Fig 1:**
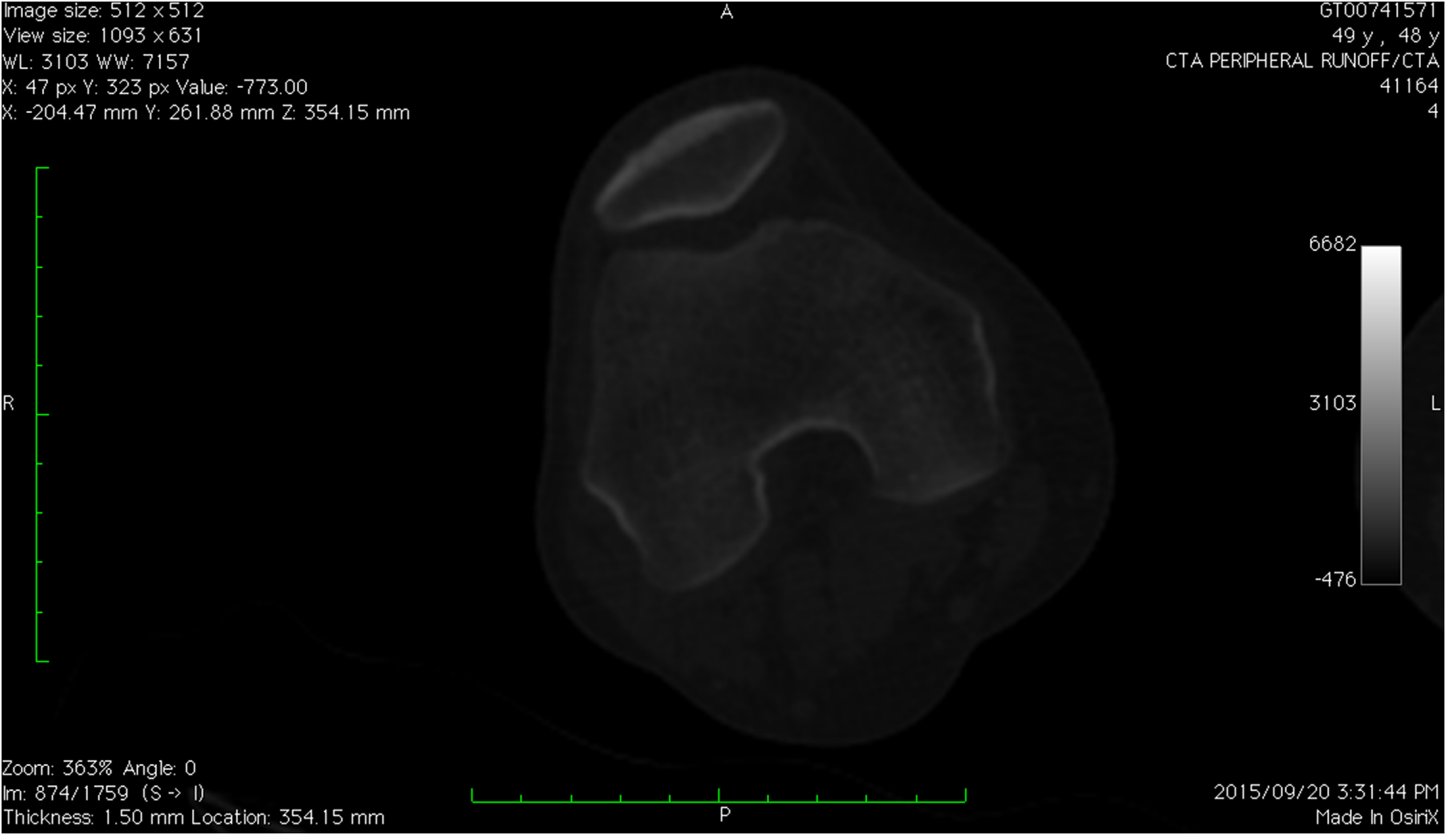
CT image of a distal femur of a living human—fresh bone. The cortex of the bone appears white with the mineralized matrix also appearing as shades of white to grey. Basic iterative reconstruction technique. Scale = 10 cm total—each bar = 1 cm.

Unlike fresh living bone, fossilized bone from Malapa has a different CT appearance. Fresh bone appears generally white on CT images with very high, positive Hounsfield unit readings (+700 to +3000 HU), reflecting the calcium content (Fig 1).

**Fig 2:**
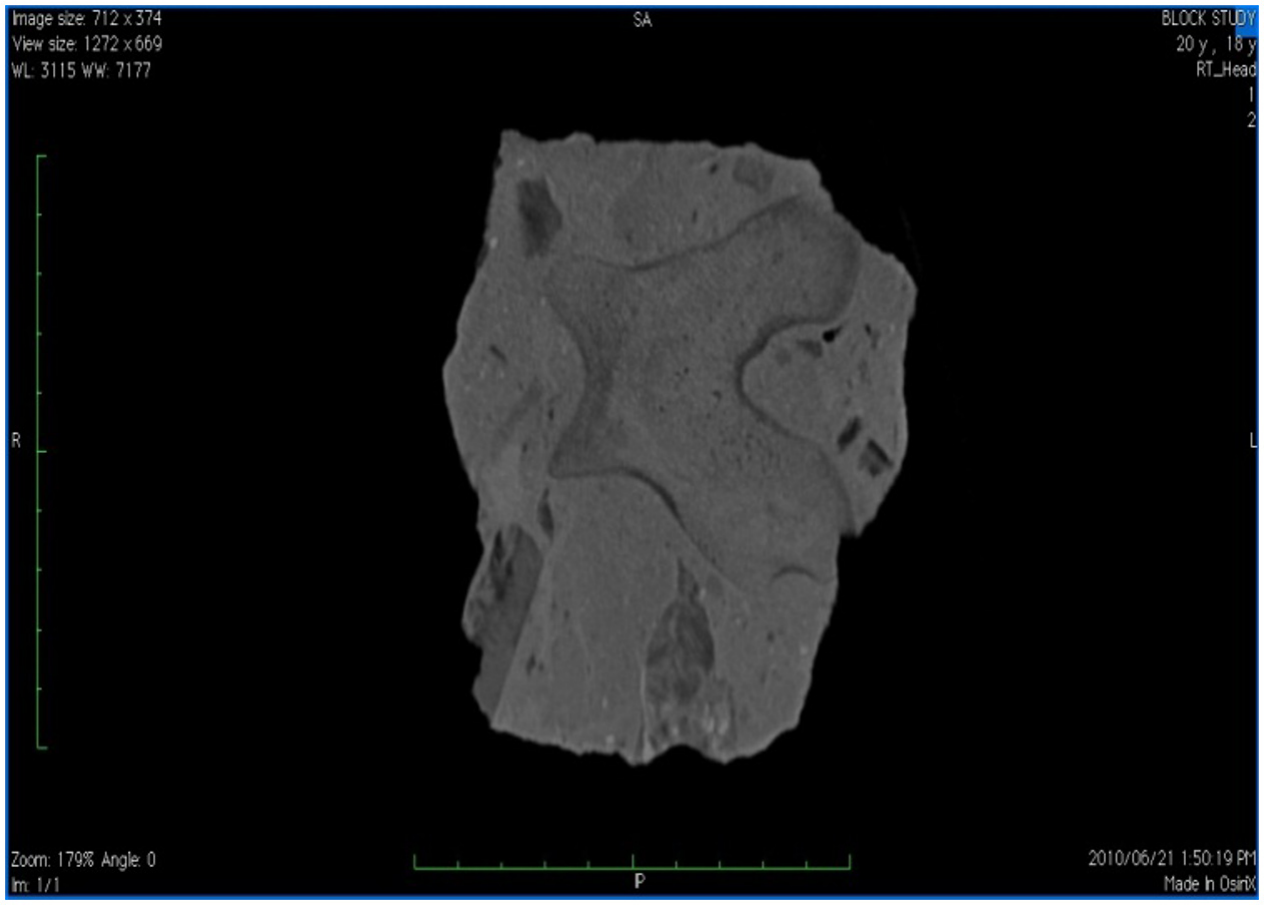
CT image of fossil of a distal bovid femur embedded entirely in matrix. The edges of the fossil appear dark grey. The central body of the fossil is of a very similar grey colour to the surrounding matrix. Small air pockets and inclusions appear black or nearly black. H70h kernel used. Scale = 10 cm total—each bar = 1 cm.

The process of fossilization involves the dissolving and replacement of the original minerals in the bone with other minerals, as well as often crystal formation within spaces and other alterations to the material [48]. This process typically results in a mineralized copy of the original object. The fossil has the same shape as the original object, but is chemically more like a rock. Some of the original bio apatite (a major bone constituent) remains, although it is saturated with silica (rock), calcium carbonate (lime) or other minerals [48]. This results in a change in density and thus appearance and Hounsfield unit reading. Fossilized bone examined in this study, typically appears grey—black on CT with Hounsfield units ranging from +300 to +1500 HU (Fig 2).

Features that were used to help differentiate possible hominin bones from other animal bones were gross skeletal anatomy (if fossils were complete enough), and cortical thickness—hominin limb bones typically having thicker cortical bone that other non—hominin bones (the thickness being relative when compared to the whole bone thickness) [49].

On analysis of the correlation of the CT findings and the manual preparation findings the following were found:

31 of the 44 blocks prepared (70.5%) had concordant findings on CT and actual preparation.
9 of the 44 cases (20.4%) showed minor variances.
4 of the 44 cases (9.1%) were discordant.

**Fig 3:**
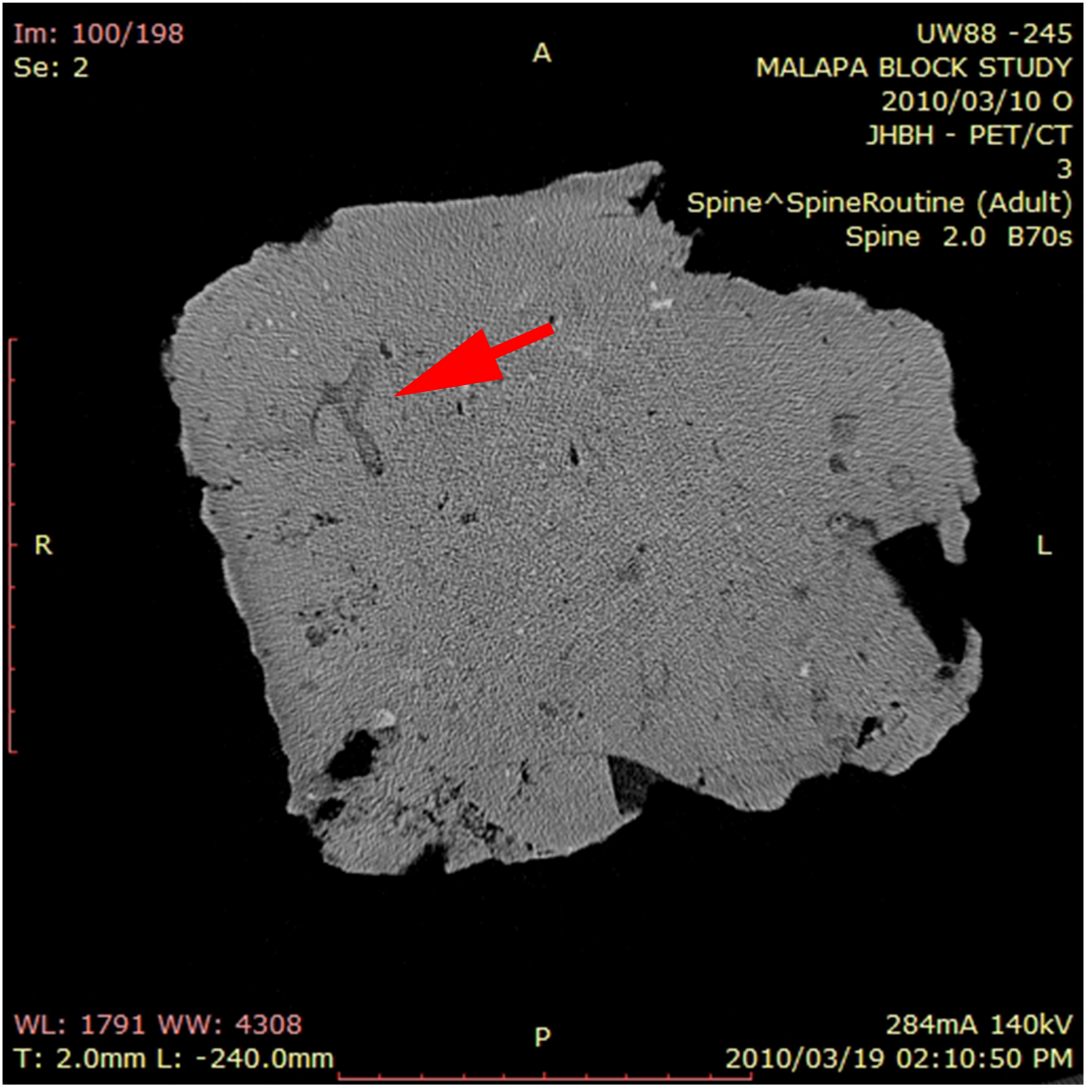
B 245 (UW 88–152)—Hominin vertebra identified on CT (arrow). Seen in sagittal projection.

**Fig 4:**
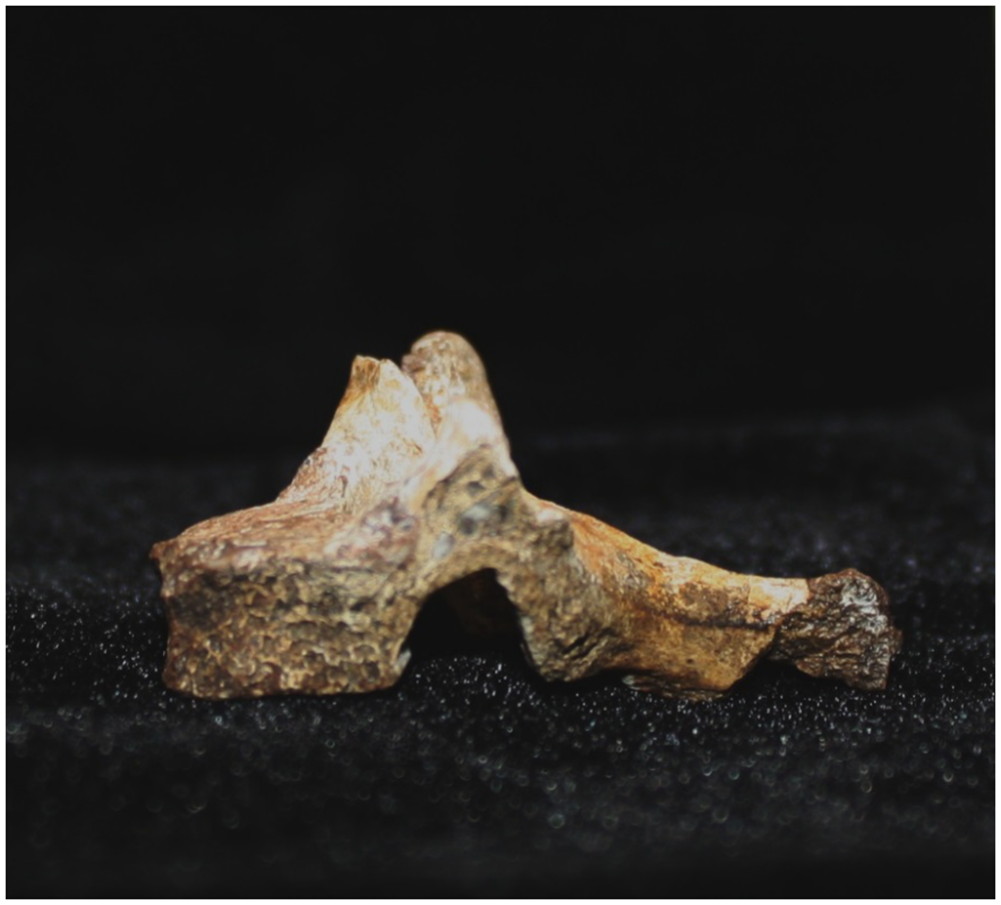
B 245 (UW88 152)–*Australopithecus sediba’s* vertebra after preparation.

Results were deemed to be concordant when the CT predictions of the identification of the bone as well as the taxonomic designation were the same as findings on manual preparation—(Figs 3 and 4). Discordant results arose when the prediction of findings from the CT had mis—identified the bones, when correlated with the actual findings post manual preparation. Minor variances were assigned when the CT had predicted the type of bone but was unable to identify the specific bone or the bone was mis-assigned taxonomically. Manual preparation then confirmed the identification of the bone. This occurred when the bone was small and fragmentary, making CT identification difficult.

Examination of the 9 cases of minor variance where CT had predicted the type of bone but could not identify further showed that this was due to several factors:

1. Where the bone was found to be crushed and fragmented, accuracy of prediction diminished with partial voluming causing erroneous appearances on CT—which were interpreted as bony anatomy.
2. CT over predicted possible hominin bones. Hominin prediction was done predominantly by assessing relative cortical thickness, and over estimation could have been due to similar density matrix making accurate cortical thickness measurement difficult.
3. The size of the fossil bones correlated with the accuracy of prediction. Bigger bones were more easily correctly identified from CT images than were small bones.

Analysis of the 4 cases where there was CT/preparation discordance showed:

1. Size of specimen again played a role in accuracy of assessment. Smaller bones were more easily missed or mis-identified.
2. Artifact contributed to poor CT- preparation correlation.
3. Complex bony architecture, such as facial bones and pelvises, were more difficult that tubular, long bones, to correctly identify.
4. Vertebrae were somewhat problematic, especially when small.
5. In 2 cases, (B164 and B286), the discordance was thought to be due to confusion in block numbering with the blocks scanned not in fact being the blocks prepared.

The CT images of the discordant cases were re-evaluated following preparation findings. Re-evaluation could not convincingly find the bones that had originally been missed, nor improve identification of visualized bones. As round shapes may be poorly seen in a single plane, careful multi planar evaluation was done, but this did not improve their identification. Visualization of each scan in multiple planes was shown to be essential for complete evaluation, as objects could sometimes be well seen in one projection, but poorly visualized in an orthogonal view [50]—tubular structure (long bones *etc*.) are well seen when viewed along their long axis but less well seen if only a transverse, short axis is viewed. These findings might suggest that the round shape is more easily missed on CT viewing, as several of the dissimilar cases, involved vertebrae or a calcaneus—the common factor here seemingly possibly the shape, although cataloguing error (where block numbers was changed between CT scanning and manual preparation) was strongly suspected in a couple of the cases.

Importantly it was noted that when viewing, the CT image should be magnified according to the object being sought. As the FOV varies according to block size, all images are initially processed to occupy the same viewer space, regardless of the blocks’ true size. Thus small objects may be very difficult to find unless the image is magnified so that the visualized size of an object mimics more closely its true size. Magnification during viewing causes enlargement of a small area by selecting that small area within the total digital field and making it cover the full display. This differs from the process of changing the FOV during image reconstruction. Reducing the FOV has the effect of increasing image detail, magnification does not do this. Without adjustment of the magnification when viewing, perspective is forgotten and smaller fossils e.g. teeth may be overlooked.

Medical CT has, over time, become standardized due to the fact that there are a limited number of different scanning subjects [50]. However, in contrast, the use of CT for palaeontological investigations requires case by case selection of scanning parameters to optimize the contrast between objects of interest whilst minimizing artifact.

Grey scale is the way in which the grey shades in a black and white image are spread. The grey shades are correlated with the digital pixel values of the image, with ranges of pixel values assigned to a certain grey shade. The CT machine will allow one to determine which matrix number should be printed as white and which should be printed as black. The numerical range between the white and black levels establishes the CT window "width". “Window width” is defined as the range of CT numbers (in Hounsfield units) included in the grey—scale display of the CT image, ranging from 1 to 2000 or 3000, depending on the type of machine. The centre of that numerical range becomes the window "level". The window width is divided by sixteen to determine the numbers which are included in each individual grey tone.

A grey scale on the display will typically contain 256 grey shades [51], whereas the Hounsfield scale has 4096 values. The range of grey scale units used during post processing varies from the Hounsfield unit scale generated by the CT machines as most medical CT systems use a 12-bit scale where 4096 values are possible, but the post processing software makes use of 16 or 64- bit scales where the range is 0–65 535 [50]. This scale does not purport to equate to the actual density of the geological materials, but allows relative comparison of densities. Ketcham et al [50] when commenting on CT use for geosciences states that *“For geological purposes*, *it is commonly more desirable to select the reconstruction parameters to maximize the CT-value contrast for each scanned object*. *This makes viewer experience with image processing essential for accurate interpretation*.*”*

The human observer can perceive no more than around 900 shades of gray. Therefore, there is an upper limit to the amount of grayscales in a medical viewing application. Display systems that are able to show 1,024 simultaneous shades of gray (10 bits) are sufficient for medical imaging. Display systems exceeding this specification will present to the human observer shades of gray that cannot be discriminated from each other anymore.[52]

The grey scale of the individual images was adjusted to reach a “best view” depiction of block contents. It has been noted by CT researchers that it is possible to set the window inappropriately and completely miss the important diagnostic information from a particular study [53]. If the window is set too wide, each grey tone will include such a wide range of tissue density that a potential object is likely to be indistinguishable from the surrounding material.

The CT reader is trained in anatomical bony recognition and has training and significant experience in digital imaging. As noted by several authors [50,54], this is essential for accurate CT prediction of fossil findings. Readers need to be familiar with the complexities of CT post processing and image manipulation as findings can be “lost” in the CT image, should the incorrect manipulations be performed or poor settings used for viewing. [54,55]

Artifact production can degrade the CT image and hinder interpretation. Modern CT machines are developed with built-in artifact reduction features, including filters, calibration correction, automatic tube current modulation and scanner software [56]. Artifact noted on the CT scans in this study was more marked in the breccia of larger volume, but interpretation was still possible. Beam hardening artifact was identified to varying degrees on the CT scans as streaking emanating from the rock’s surface and through the rock. Willis *et al* [57] showed that the higher photon attenuation and irregular shape of fossilized material lead to severe streak artifact resulting from abrupt changes in X-ray transmission intensity across an object and was often associated with long straight edges of high attenuation material. Scanning at a higher kV results in a harder X-ray beam, and thus less beam hardening artifacts, hence the choice of the maximum KVp of 140 on the medical scanner.

Of note, was that no hominin fossils were missed on CT predictions, in blocks prepared. This may be due to the general relative thicker cortices [49] of hominin bones over other animals, thus facilitating their identification even when round in shape. Not only was CT convincingly able to identify the presence of fossil bone, but good visualization allowed good predictive identification and characterization. CT scans offer a quick and non-destructive method of imaging. Data is obtained in a digital format that allows 3 dimensional representations of an object to be created. Post processing of this data allows reconstructions, measurements and a variety of analyses to be performed.

There are however limitations to the use of CT in palaeontological research, many of these are being overcome as hardware and software improvements occur and advancements in technology are made. The CT image can be manipulated and visualized depending on the required application. Often 2 D analyses along orthogonal planes are sufficient for skeletal structures, but additional information can be gained from 3 D image reconstructions. Traditionally these 3D reconstructions are calculated from CT values based on CT grey scale numbers. If quantitative measurements are needed from the CT data, segmentation techniques are often necessary to separate features of interest based on criteria other than CT values, as use of CT numbers may be complicated by partial volume averaging effects [58]. Manipulation of CT parameters at the time of scanning, to lessen imaging artifact, can hinder precise image acquisition [59]. Partial voluming and limits in spatial resolution are important constraints of CT [23]. Scientists have tested and validated the accuracy of these latter two constraints as regards the accuracy and reproducibility of measurements and the definition of landmark coordinates [60–62].

The use of medical CT was specifically investigated, as oppose to industrial CT with micro CT capabilities or other scanning technologies (e.g. synchrotron) for the following reasons:

- Industrial micro CT is presently not readily available in South Africa [55], scanning times are longer than medical CT (hours versus seconds on a medical scanner) [63] and size and weight restrictions on these micro CT machines are significantly more limiting than on medical CT scanners. (The micro CT at the University of Witwatersrand takes a maximum diameter of +/- 20 cm and a weight of 50–60 kg, whereas medical CT allows diameter of 80–90 cm and weights of between 200–300 kg).
- Synchrotron scanners are very scarce, immensely costly and very time consuming.
- Both micro CT and synchrotron imaging generate very large data sets, necessitating dedicated computers and software with large data handling capabilities. These are expensive and not as freely available as ordinary laptop or desktop computers that can handle the DICOM data generated from the medical scanner. In addition, medical CT data can be analyzed with software that is free.

## Conclusions

In 2009, Wu [4] stated “*The suitability of medical CT for the study of hominin fossils is limited by its low X-ray dosage that is unable to penetrate highly mineralized and matrix-filled specimens*.” Scanning of 109 matrix fossil- bearing rocks from the site of Malapa yielded CT images of a quality that, coupled with modern software post processing programmes and a suitably trained and experienced reader, allowed for the accurate prediction of the fossil contents of the blocks.

Medical CT scanners are shown by this study to be capable of producing images that allow for the accurate identification and often characterization of fossils. The correlation of these predicted findings with the actual findings post manual preparation is sufficiently concordant to change the traditional course of the handling of fossil bearing blocks. In order to maximize the use of limited resources and manual preparatory skills as well as to curtail costs, this research suggests that prior to manual preparation, blocks should undergo scanning with medical CT scanners and “virtual” assessment of contents should be undertaken by suitably qualified individuals to allow for prioritization of rocks for manual preparation. Knowledge of bony as well as radiological anatomy is deemed essential for accurate interpretation of findings, as is familiarity and experience with digital imaging techniques, their production limitations and pit falls of post processing manipulation.

For the first time in South African palaeoanthropological work hominin fossils have been imaged within their matrix before any preparation had been performed. The relationships of all the bones could be eloquently demonstrated on post- processing of the CT images. This afforded scientists the unique opportunity to, up front, assess and plan the further investigation and preparation of this block—something never achieved in South Africa before.

The results of this study have shown conclusively the viability and value of the use of medical CT imaging to assess possible fossil-containing rocks for fossil remains. It has also demonstrated that there is considerable advantage to being able to know the contents of a rock ahead of costly, time-consuming “blind” manual preparation. This allows decisions to be made as regards the most efficient use of resources, manpower and allocation of funds, in addition to allowing planning of the course of action for each fossil. Information can be extracted without damage to the matrix and hence allows the potential for preservation of these remains for future generations of scientists, ensuring that as technology advances, enough direct physical evidence has been left behind on which to apply new methods of analysis in the future.
